# A novel form of PCD that impacts nodal, but not tracheal cilia

**DOI:** 10.1186/2046-2530-4-S1-O15

**Published:** 2015-07-13

**Authors:** J Keynton, E Adams, K Riley, N Powles-Glover, K Shinohara, J Lucas, P Lackie, D Norris

**Affiliations:** 1 MRC Harwell, Oxford, UK; 2 Southampton General Hospital, University of Southampton, Southampton, UK; 3 Osaka University, Osaka, Japan

## 

Motile cilia, in the embryonic node, drive a leftward fluid flow (termed nodal flow) that establishes the left-right axis. We identified *lrm5 *in a genetic screen for mouse left-right patterning mutants - the embryos exhibited disturbed situs. Mapping and sequencing revealed a novel mutation in the axonemal dynein heavy chain locus *Dnah11*: loss of function gives rise to immotile cilia in mice; many human primary ciliary dyskinesia (PCD) patients with DNAH11 mutations have hyper-motile cilia. To our surprise, unlike the previously characterised *Dnah11^iv ^*mutant, *lrm5 *tracheal ciliary beat frequency (CBF) was normal. However, the number of *lrm5 *homozygotes at weaning was lower than Mendelian ratios would predict. Age of death analysis identified a 50% reduction in embryos between E14.5 and E15.5, consistent with death from embryonic cardiac failure. Expression analysis of early molecular markers of left-right asymmetry revealed randomised or bilateral activation of the normally left-sided Nodal Cascade. As this suggested an early, primary patterning defect, we analysed nodal flow by particle image velocimetry (PIV); rather than the wild-type leftward flow, or the absent flow in *Dnah11^iv^*, we observed a chaotic fluid flow in *lrm5 *nodes. We therefore assessed nodal CBF and ciliary amplitude by DIC microscopy; *lrm5 *cilia beat at 1.5x normal frequency, but with an abnormal motion. In summary, *lrm5 *is a novel form of PCD, impacting nodal but not tracheal ciliary beating. We would predict that equivalent mutations in humans might underlie situs defects and congenital heart disease in the absence of respiratory disease.

